# Mechanical Dentistry

**Published:** 1878-05

**Authors:** J. B. Tullis


### MECHANICAL DENTISTRY.

BY J. B. TULLIS, D. D. S.

This branch of dentistry at the present day stands very prominently before the profession and the public. As to its origin very little is known, at least I have not seen much in reference to it, by whom or where it was first introduced into practice. I presume that like many other things of utility it was first incidently applied to meet some special case, and afterwards as cirsumstances demanded, experiments were made until it has attained its present attitude in the dental profession. When none but metallic base was used, there was not so much done as now in this department. It required some knowledge of, and skill in working metals to construct a plate that could be used comfortably and utilized in the mastication of food. There was a greater effort then made to save the natural teeth by the profession at large than now. This was a great blessing to the people, saving their teeth from the wholesale slaughter as is now practiced by many. While it is a blessing in some cases it is an evil in many others. In consequence of its abuse I am almost inclined to the opinion it was a misfortune it ever became a branch of dentistry proper. Before its introduction, so far as known, I have no doubt more pains was taken to save the natural teeth then, than now in proportion to the knowledge possessed by practitioners. Then full sets of artificial teeth were seldom seen. Now the easy manipulation of rubber and its cheapness, has and does induce many an inferior operator to insist on his patient having all the teeth removed, and have some made that will never ache or decay. In order to be relieved from these calamities, they submit. Good, defective and very defective teeth shared alike in the wholesale slaughter of the tooth family, and in due time these parties exhibit the wonderful skill of Dr. ———, the renowned dentist of the world, he made my teeth. I advise all to have their teeth "pulled," as the (renowned dentist says plugging does but little good to save teeth. That settles the question beyond controversy.

There are men, who on rubber put up a good plate, and at the same time know but little of the science of dentistry, or its import professionally. A knowledge of the mechanical department of dentistry by observation and experience does not constitute the man a dentist. There are some experts in the operative, mechanical and surgical departments of dentistry who are not well informed in the general science of dentistry. A knowledge of all is necessary, not that he shall practice all of necessity. If it were practicable a division of the operative and mechanical branches should be made. In large cities this might be done, but in small towns and rural districts it is impracticable. The cheapness of mechanical dentistry now brings a class of men before the public in a false light, because a man can construct a plate of rubber he is recognized as a dentist, equal or superior to the man who by study has qualified himself to meet the requirements of the dentist. "Pulling teeth and making teeth" is not all that a man should know to be a dentist in fact.

The want of information on the part of the people subjects them an easy prey for charlatans, and they use their opportunities freely. The better informed members meet these things all the while, I heard a man say a quack could do as well as any, and there should be no prescribing men in a free country. If this be true our colleges are public farces, and educated men farces, especially in a profession—the dental profession.

Let the better informed, labor to build up and educate the people, and the worthy will be rewarded, and our profession will move forward to the front and be hailed with delight by an appreciative public. Conservative dentistry should be fostered by all the good in the profession, and we will have less need for the mechanical department.

